# Old Age and Death

**Published:** 1920-11-27

**Authors:** 


### Old Age and Death.

### PROBLEMS OF MODERN CIVILISATION.

A writer in the daily press bids us be of good cheer. There is no reason, he says, why we should not live for ever. In reply to the two questions, "What exactly is old age, and what is the natural limit to the life of man?" modern science returns the simple answer, that it is impossible to say that either one or the other really exists as the outcome of natural laws. In the human body there is no inevitable process of decay at work, gradually encompassing the paralysis of the individual by a natural law of death. That animal tissue is capable of extended life has been proved beyond doubt by experiments in which portions of organs have been removed from animal bodies and placed in nutrient solutions; where they thrive and live an independent existence, showing none of the signs of an old-age stealing upon them. The natural conclusion of this pleasing argument is that death is an unavoidable accident. But what gives the argument a touch of academic remoteness is the fact that the accident invariably occurs. At the same time it is a good viewpoint to take, both for the profession of healing and for mankind at large—that *premature* death can be avoided, and ought to be avoided. The whole basis of the modern theory of the service of public health is that it should aim to establish conditions which will prevent illness and avoid death, not the death which comes after a long and vigorous life, but the breakdown of the individual before the allotted span or at the very outset of life. The modern organisa-

**THE PUBLIC WELL-BEING**—*(continued)*.

tion of public health in a civilised community is engaged in the tremendous struggle of trying to create immunity from the complicated effects of an increasingly artificial existence; in other words, to fight the problems of science with the solutions of science. No man can predict with confidence how that struggle will eventuate. Will the strain of living shorten life in the future, or will preventive measures on a gigantic and well-organised scale compensate the strain and give a longer life? Will longer life be worth while? Perhaps the last question is less important, because, if the standard of living gets so low, or the miseries of life become so generally acute that the generality of mankind finds life not worth the living, mankind will automatically give up the attempt to prolong it. We know now that, with conspicuous exceptions, those who preserve something of their youthful vigour to an advanced age are persons of an even and placid temperament, who in the midst of anxieties, fears, ferments of all kinds, have the inherent capacity to remain calm and undisturbed. The placid temperament, plus an intelligent interest in the common things of life, is the surest index to longevity. It will be extremely interesting in the next ten or twenty years to study closely the mortality statistics of any civilised country in order to ascertain how far that temperament is being destroyed by the physical and mental demands of modern existence, or how far the efforts born of an enlightened knowledge of communal health problems will provide the counter-acting influence. As a physician and a visionary said the other day, the best hope for the human race lies in the discovery of a universal germicide, under perfect control, and, therefore, capable of infinitely exact application. It seems a good deal to hope for.

